# A Two-Phase Expansion Protocol Combining Interleukin (IL)-15 and IL-21 Improves Natural Killer Cell Proliferation and Cytotoxicity against Rhabdomyosarcoma

**DOI:** 10.3389/fimmu.2017.00676

**Published:** 2017-06-12

**Authors:** Juliane Wagner, Viktoria Pfannenstiel, Anja Waldmann, Judith W. J. Bergs, Boris Brill, Sabine Huenecke, Thomas Klingebiel, Franz Rödel, Christian J. Buchholz, Winfried S. Wels, Peter Bader, Evelyn Ullrich

**Affiliations:** ^1^Children’s Hospital, Goethe University, Frankfurt am Main, Germany; ^2^Division for Stem Cell Transplantation and Immunology, Department for Children and Adolescents Medicine, Hospital of the Goethe University Frankfurt, Frankfurt am Main, Germany; ^3^LOEWE Center for Cell and Gene Therapy, Goethe University, Frankfurt am Main, Germany; ^4^Georg-Speyer-Haus, Institute for Tumor Biology and Experimental Therapy, Frankfurt am Main, Germany; ^5^Department of Radiotherapy and Oncology, Goethe University, Frankfurt am Main, Germany; ^6^German Cancer Consortium (DKTK), Partner Site Frankfurt/Mainz, Frankfurt am Main, Germany; ^7^German Cancer Research Center (DKFZ), Heidelberg, Germany; ^8^German Cancer Consortium (DKTK), Partner Site Heidelberg, Heidelberg, Germany; ^9^Molecular Biotechnology and Gene Therapy, Paul-Ehrlich-Institut, Langen, Germany

**Keywords:** natural killer cells, radiotherapy, rhabdomyosarcoma, RH30 cells, RD cells, interleukin-15, interleukin-21

## Abstract

Rhabdomyosarcoma (RMS) is the most common soft tissue malignancy in children. Despite intensive research in recent decades the prognosis for patients with metastatic or relapsed diseases has hardly improved. New therapeutic concepts in anti-tumor therapy aim to modulate the patient’s immune system to increase its aggressiveness or targeted effects toward tumor cells. Besides surgery, radiotherapy and chemotherapy, immune activation by direct application of cytokines, antibodies or adoptive cell therapy are promising approaches. In the last years, adoptive transfer of natural killer (NK) cells came into the focus of translational medicine, because of their high cytotoxic potential against transformed malignant cells. A main challenge of NK cell therapy is that it requires a high amount of functional NK cells. Therefore, *ex vivo* NK cell expansion protocols are currently being developed. Many culturing strategies are based on the addition of feeder or accessory cells, which need to be removed prior to the clinical application of the final NK cell product. In this study, we addressed feeder cell-free expansion methods using common γ-chain cytokines, especially IL-15 and IL-21. Our results demonstrated high potential of IL-15 for NK cell expansion, while IL-21 triggered NK cell maturation and functionality. Hence, we established a two-phase expansion protocol with IL-15 to induce an early NK cell expansion, followed by short exposure to IL-21 that boosted the cytotoxic activity of NK cells against RMS cells. Further functional analyses revealed enhanced degranulation and secretion of pro-inflammatory cytokines such as interferon-γ and tumor necrosis factor-α. In a proof of concept *in vivo* study, we also observed a therapeutic effect of adoptively transferred IL-15 expanded and IL-21 boosted NK cells in combination with image guided high precision radiation therapy using a luciferase-transduced RMS xenograft model. In summary, this two-phased feeder cell-free *ex vivo* culturing protocol combined efficient expansion and high cytolytic functionality of NK cells for treatment of radiation-resistant RMS.

## Introduction

With their ability to detect and directly destroy virally infected or malignant cells, natural killer (NK) cells form an important part of the first line defense of the immune system. They can be activated rapidly *via* germ-line encoded receptors that recognize the presence of stress ligands or absence of self-antigens on target cells (1–5).

*In vivo* development and survival of NK cells require cytokines (6–8). In this context, cytokines have been shown to activate NK cells potently during *ex vivo* expansion (9–12). The group of common γ-chain receptor cytokines encompassing interleukin (IL)-2, IL-4, IL-9, IL-15, and IL-21 has been studied intensively over the recent years. IL-2 and IL-15 have similar impacts on NK cells (13, 14). However, direct injection of IL-2 has been shown to be accompanied by severe side effects, such as vascular leak syndrome, activation-induced cell death, and strong induction of regulatory CD4^pos^ T cells, which did not occur after IL-15 administration (15, 16).

More recently, research has been focusing on IL-21 biology, but its effects on NK cell development are controversially discussed. IL-21 is known to be involved in the development and proliferation of NK cells from progenitor cells (17) and to induce receptor expression (18), interferon (IFN)-γ secretion and cytotoxicity (19). Conversely, IL-21 has also been reported to trigger apoptosis and to diminish IL-15-based benefits (20–22). These less favorable effects may be ascribed to the variability of experimental designs such as timing, cytokine concentration, additives, or accessory cells in culture as well as the developmental or maturation state and origin of NK cells. Of note, positive effects have been reported mostly upon cultivation of NK cells in the presence of auxiliary cells such as other peripheral blood mononuclear cells (PBMCs) (23), genetically modified feeder cells equipped with membrane-bound IL-21 (24, 25), or feeder-cell particles (26). The downside of these protocols is the necessity of elimination of hazardous cells, such as possibly graft-versus-host-disease (GvHD)-triggering cells or tumor-derived feeder cells, that might induce harmful side-effects *in vivo*. On the contrary, safer expansion strategies based on the exclusive application of cytokines, result in much lower absolute NK cell numbers (27). Thus, risk-free protocols for efficient expansion of functional NK cells are urgently needed.

Immune cell therapy is an effective anti-cancer strategy and hematopoietic stem cell transplantation (HSCT) has been shown to positively influence the outcome of patients with different hematologic diseases (28, 29). However, studies using HSCT did not achieve satisfactory improvement against high-risk rhabdomyosarcoma (RMS) (30–34). RMS is a rare malignant disease but the most common soft tissue cancer in children. The outcome of treatment for patients with stage IV RMS, relapsed or metastatic diseases arising from RMS, has scarcely improved during recent decades and, in general, is unfortunately considered to be poor even upon combination of surgery, chemotherapy, radiotherapy (RT), and HSCT (35, 36).

Natural killer cells are considered to potently initiate graft-versus-tumor (GvT) effects without provoking, but even preventing GvHD (37–41), a possible risk of HSCT (42–44).

Here, we present a two-phase protocol that combines IL-15-triggered NK cell expansion with an IL-21 boost to exert the stimulatory effects of both cytokines. To avoid contamination by other cell types, we employed enriched NK cells and circumvented the addition of any accessory or feeder cells. The NK cell product was characterized intensively in terms of proliferation, phenotype, and functionality. Finally, IL-15-expanded and IL-21-boosted NK cell products from different human donors were used for combined adoptive immune cell and radiation therapy in a xenograft model of RMS.

## Materials and Methods

### Purification of Primary Human NK Cells

This study was approved (approval no. 329/10) by the Ethics Committee of the Goethe University Frankfurt (Frankfurt, Germany) and was performed in accordance with the Declaration of Helsinki with written informed consent given by every participant. NK cells were isolated from freshly generated donor buffy coats provided by the German Red Cross Blood Donation Service (DRK-Blutspendedienst Baden-Württemberg-Hessen, Frankfurt, Germany), using immunomagnetic negative selection (EasySep™ Human NK Cell Enrichment kit, StemCell Technologies, Vancouver, BC, Canada) according to the manufacturer instructions.

Briefly, PBMCs obtained from buffy coats by density gradient centrifugation were diluted to a final concentration of 100 × 10^6^ cells/mL. Then, 50 µL of enrichment cocktail were applied per milliliter cell suspension and incubated for 10 min. Subsequently, 100 µL of microbead suspension were added and incubated for another 5 min. After incubation for 2.5 min in “The Big Easy” EasySep™ Magnet, the NK cell-enriched suspension was decanted into a new tube.

### Cultivation of Primary Cells and Cell Lines

Purified primary NK cells were cultured at a concentration of 2 × 10^6^ cells/mL in X-VIVO 10 medium (Lonza Group Ltd., Basel, CH) supplemented with 5% heat inactivated human fresh frozen plasma (FFP; provided by DRK-Blutspendedienst Baden-Württemberg-Hessen, Frankfurt, Germany) and 100 U/mL penicillin and 100 µg/mL streptomycin (Gibco, New York, NY, USA). Correlating to the batch, the cells were provided with IL-2 (ProleukinS, Novartis Pharmaceuticals, Horsham, UK), 100 U/mL (IL-2^100^) or 1,000 U/mL (IL-2^1000^), 10 ng/mL IL-15 (IL-15), 25 ng/mL IL-21 (both PeproTech, Rocky Hill, CT, USA) (IL-21) or combinations of those (IL-2^100^ + 15, IL-2^100^ + 15 + 21, IL-15 + 21). Every 3–4 days, half of the medium was replaced by fresh medium containing the corresponding cytokines or combinations. One batch was treated with IL-15 and boosted with IL-21 only 3–4 days before harvest and analysis (IL-15 + 21*_boost_*).

Chronic myologenous erythroleukemia cell line K562 and RMS cell lines RH30 and RD (45) were purchased from the American Type Culture Collection (Manassas, VA, USA). Cells were maintained in Roswell Park Memorial Institute (RPMI) 1640 medium supplemented with 10% heat inactivated fetal calf serum (Invitrogen, Paisley, UK), 100 U/mL penicillin and 100 µg/mL streptomycin. The cells were splitted twice a week.

GFP/luciferase-expressing RD cells (RD^GFP/Luc^) were generated *via* lentiviral transduction using vector particles pseudotyped with vesicular stomatitis virus G protein that were produced using the transfer plasmid pSEW-luc2, which encodes firefly luciferase and enhanced green fluorescent protein linked *via* a 2A peptide (46). GFP positive cells were enriched by fluorescence activated cell sorting (FACS) using a FACSAria II™ device (BD Biosciences, San Jose, CA, USA). Culture conditions for transduced cells were the same as for non-transduced cells.

### Flow Cytometry

In order to check the quality of enriched NK cells and to monitor the phenotype of *ex vivo* expanded NK cells, samples were analyzed with a FACSCanto 10c™ system (BD Biosciences). Post-harvesting cells were resuspended in FACS buffer containing CellWASH (BD Biosciences), 0.5% bovine serum albumin (Sigma Aldrich, Taufkirchen, Germany) and 0.01% NaN_3_ (0.1 M, Sigma Aldrich).

Intracellular staining was accomplished using formaldehyde (AppliChem GmbH, Darmstadt, Germany) for fixation and 90% methanol for membrane perforation.

The following antibodies were used: CD3-APC (#UCHT1), TRAIL-R-APC [#DJR2-4(7-8)], FAS-BV421 (#DX2), CD56-FITC (clone #HCD56), FAS-L-PE (#NOK-1), TRAIL-PE (#RIK-2), CD19-PerCP (#HIB19), CD16-PE/Cy7 (#3G8) all from Biolegend (San Diego, CA, USA); CD3-V450 (#UCHT1), CD19-V450 (#HIB19), CD14-V450 (#MφP9), CD45-BV510 (#HI30), NKp30-AF488 (#P30-15), DNAM-1-FITC (#DX11), NKp44-PE (#P44-8.1) CD45-APC (#2D1), CD137/4-1BB-APC (#4B4-1), CD107a-APC/H7 (#H4A3), IFN-γ-FITC (#B27), pAKT-AF647 (#F29-763), pERK1/2-AF647 (#20A), from BD Biosciences; CD56-APC/AF700, NKG2D-APC (#ON72), CD11a/LFA-1-FITC (#25.3) from Beckman Coulter Immunotech (Brea, CA, USA); CD45-PE (#HI30) from Invitrogen (Carslbad, CA, USA); and NKp46-APC (#9E2), KIR2D-FITC (#NKVFS1), CD158e/k-PE (#5.133), NKG2A-APC (#Z199) from Miltenyi Biotec (Bergisch-Gladbach, Germany). Depending on the panel Zombie Violet Fixable Viability Kit (BioLegend), 7AAD (BD Biosciences) or strongly diluted DAPI were used for live/death discrimination.

Data were acquired on a FACSCanto 10c™ instrument (BD Biosciences, San Jose, CA, USA) and analyzed using Flowjo (Tree Star Inc., Ashland, OR, USA).

### Cytotoxicity Assay

To investigate the killing capacity of *ex vivo* expanded NK cells a FACS-based cytotoxicity assay was employed. NK effector cells were harvested after 6 days of cytokine stimulation. Target cells were harvested and stained for 5 min with Celltrace CFSE (Molecular Probes, Eugene, OR, USA) in a final concentration of 5 µM. After all cells had been washed with Dulbecco’s Phosphate Buffered Saline (DPBS, Gibco), they were resuspended in X-VIVO 10 medium supplemented with 5% heat inactivated human FFP, 100 U/mL penicillin and 100 µg/mL streptomycin. NK cells and target cells were combined in a U-bottom 96-well plate at effector to target (E:T) ratios of 1:1, 5:1, and 10:1, adjusted to 25,000 target cells per well in a total volume of 200 µL. After 5 h of co-incubation the supernatant was removed, cells were harvested and resuspended in a 1:6,000 dilution of DAPI for live/death discrimination. From each well, the same amount of target cells was acquired using a FACSCanto 10c™ device. Samples exclusively containing target cells served as spontaneous lysis controls. Spontaneous lysis was subtracted from each sample to obtain specific lysis values. All experiments were conducted in triplicates for each NK cell donor.

To address additional antibody-dependent cellular cytotoxicity (ADCC) by NK cells, in a separate experiment, 10 µg/mL of anti-ErbB2 antibody Trastuzumab (Herceptin, ROCHE, Mannheim, Germany) were added and cytotoxicity compared to NK cell cytotoxicity in the absence of Trastuzumab.

Conjugation capacity of stimulated NK cells was addressed by staining NK cells with Celltrace CFSE, while target cells were stained with Celltrace Calcein Violet AM for 20 min at 4°C. After intensive washing, effector and target cells were co-incubated for 0–90 min, then shortly vortexed and fixed with 1–2% formaldehyde. Flow cytometry data were acquired on a FACSCanto 10c™ instrument.

Due to limited availability of NK cell numbers, cytotoxicity and conjugation assays were performed with cells from other donors than were used for proliferation assays.

### Functional Activity and Degranulation Assay

Degranulation potential of cytokine stimulated NK cells was assessed as described (47), with cells harvested on day 6 of cultivation. Cells were washed and resuspended in fresh X-VIVO 10 medium supplemented with 5% heat-inactivated human FFP, 100 U/mL penicillin, and 100 µg/mL streptomycin. After 1 h, cells were incubated with anti-human CD107a, followed by an additional hour of incubation with GolgiStop™ (BD Biosciences). Cells were washed, blocked with human IgG and stained with Zombie Violet™ Fixable Viability Kit for live/death discrimination. Post washing, cells were stained for CD45, CD56, and CD16, fixed with formaldehyde solution (2% final concentration) and permeabilized with saponin buffer [0.2% saponin, 1% bovine serum albumin (both Sigma Aldrich) in DPBS]. In the end, cells were stained intracellularly with anti-human IFN-γ, washed, and measured by flow cytometry.

For Phosflow analysis freshly harvested cells were stained on the surface for CD45, CD3, and CD56, fixed and permeablilized with 90% methanol and intracellularly stained for pAKT, pERK1/2, and pMAPK. Flow cytometry data acquisition was performed on a FACSCanto 10c™ instrument.

Due to limited availability of NK cells numbers, degranulation assays were performed with cell preparations from different donors than proliferation or cytotoxicity assays.

### Immunoblot Analyses

Western blotting was deployed for the assessment of production and release of apoptosis-mediating perforin and granzyme B. NK cells and culture supernatants were harvested on day 6 of cytokine stimulation. Cells were lysed using RIPA buffer supplemented with cOmplete™ Protease Inhibitor Cocktail (ROCHE) followed by sonification. Protein concentrations were determined *via* Bradford assay (Protein Assay Dye Reagent Concentrate, Bio-Rad, Munich, Germany). Separation of proteins was accomplished by SDS-PAGE followed by semi-dry blotting onto polyvinylidenfluoride membranes. Before antibody application, membranes were blocked with 5% skim milk powder in DPBS. Mouse monoclonal antibodies against human perforin (1:1,000, LifeSpan BioSciences, Seattle, WA, USA), granzyme B (1:200, #2C5, Santa Cruz, Heidelberg, Germany) and a rabbit antibody against human γ-tubulin (1:2,000, Sigma Aldrich) were used as primary antibodies. HRP conjugated rabbit anti-mouse IgG (1:15,000, Sigma Aldrich) and goat-anti rabbit (1:16,000, Sigma Aldrich) served as secondary antibodies. The antigen–antibody complexes were detected using an ECL-chemiluminescence system (Pierce™ ECL, Thermo Scientific, Waltham, MA, USA) according to the manufacturer’s instructions, and visualized using X-ray films (Fujifilm, Tokyo, Japan).

Due to limited availability of NK cell numbers, immunoblot assays were performed with cells from other donors than used for proliferation or functional assays.

### Cytometric Bead Array

Cytokine secretion was examined by cytometric bead array analyses (CBA) on supernatants of stimulated NK cells on the sixth day of cultivation using BD CBA Flex Sets for IFN-γ, tumor necrosis factor (TNF)-α, macrophage inflammatory protein (MIP)-1α, monocyte chemoattractant protein (MCP)-1, IL-8, IL-10, and granulocyte-macrophage colony-stimulating factor (GM-CSF) (BD Biosciences). The tests were performed according to the manufacturer’s instructions using a mixture of PE-conjugated antibodies against the cytokines listed above. Data were acquired with the BD FACSVerse™ Bioanalyzer and were quantitated using the FCAP Array™ software (v3.0.1; BD Biosciences).

### Murine RMS Xenograft Model and Treatment Protocol

The *in vivo* experiments were approved by the government committee (Regierungspräsidium Darmstadt, Darmstadt, Germany) and were conducted in accordance to the requirements of the German Animal Welfare Act.

Female, 12- to 16-week-old NOD/SCID/IL-2Rγc^−/−^ mice (NSG) mice were injected subcutaneously with 10^5^ luciferase expressing RD^GFP/Luc^ cells. After visual detection of tumor nodes about 3 weeks post-cell injection, mice were imaged by performing a Cone-Beam CT (CBCT) operating at 65 kV, 0.5 mA and irradiated while immobilized with 2.5% isoflurane anesthesia (AbbVie, Wiesbaden, Germany) using a Small Animal Radiation Research Platform (SARRP, Xstrahl Ltd., Camberley, UK). CBCT images were transferred to MuriPlan™ Software and individual isocenters were selected for targeted radiation therapy applying a two-field geometry. Fractionated single doses of 2.5 Gy using a 1–10-mm collimated beam operating at 175 kV, 15 mA were applied four times a week to reach a total dose of 27.5 Gy. Post-termination of RT, adoptive transfer of IL-15 expanded and IL-21 boosted NK cells was accomplished in three injections, once a week. NK cells were purified from 11 buffy coats as described above and maintained separately during cultivation in 25 cm^2^ suspension cell culture flasks (Cellstar, Frickenhausen, Germany) using the IL-15 + 21*_boost_* protocol as described before. After 10–11, 17–18, and 24–25 days of *ex vivo* expansion and stimulation, NK cells were harvested, washed, and pooled. The NK cells were injected intravenously *via* the tail vein with 10^7^ NK cells in a total volume of 100 µL per mouse. Due to limited availability of NK cell numbers, *in vivo* application was performed with cells from other donors than used for proliferation or functional assays.

Tumor growth was monitored by caliper measurements and bioluminescence imaging (BLI) using an IVIS Lumina II system (Perkin Elmer, Waltham, MA, USA). For the latter method, mice were anesthetized by isoflurane inhalation and subcutaneously injected with 150 µg of *in vivo* grade VivoGlo™ luciferin (Promega, Madison, WI, USA) dissolved in 100 µL DPBS per mouse. Images were acquired after an incubation time of 15 min. BLI data analysis was performed using Living Image^®^ software (Perkin Elmer).

### Statistical Analyses

Results were analyzed using repeated measures one-way ANOVA with the Geisser–Greenhouse correction. For *in vivo* experiments, two-way ANOVA was used to compare the tumor-growth curves of different treatment groups. Statistical calculations were performed using GraphPad Prism v6 (GraphPad, La Jolla, CA, USA), and *p* values <0.05 were considered statistically significant.

## Results

### IL-15-Driven NK Cell Expansion Is Not Impaired by a Short-term Boost with IL-21

**Figure 1:**
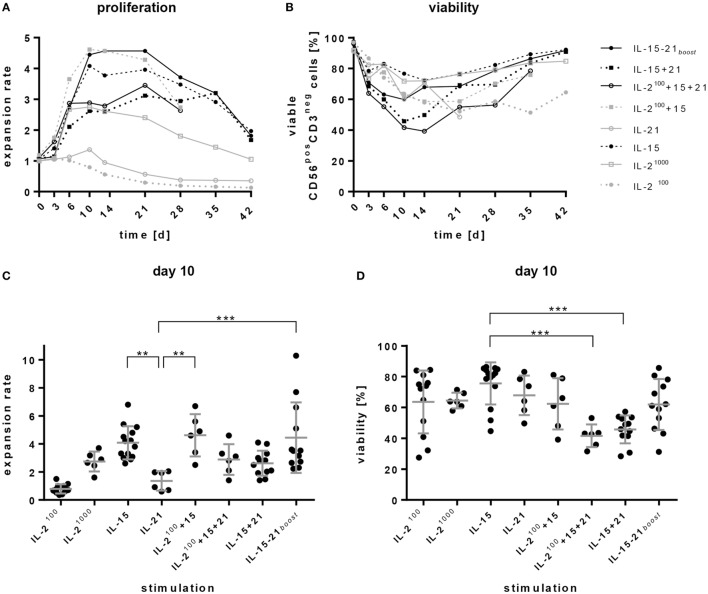
Expansion and viability of natural killer (NK) cell products. Purified CD56^pos^CD3^neg^ cells were cultivated for 4–6 weeks in the presence of different cytokines or cytokine combinations. **(A)** Proliferation is presented as expansion rate. Here, the mean values of 6–14 donors are shown over time. **(B)** Viability of CD56^pos^CD3^neg^ NK cells was analyzed by DAPI staining at the indicated time points. **(C,D)** show the donor dependent distribution of values on day 10 for proliferation and viability, respectively. Lines represent mean values and SDs. Significant differences are indicated by asterisks (**p < 0.01, ***p < 0.005, one-way ANOVA). Although not indicated in the graph, IL-2^100^ and IL-21 alone induced significantly smaller expansion rates than all other protocols.

The primary goal of *ex vivo* expansion of NK cells is to yield high cell numbers for adoptive transfer with an appropriate quality and optimal cytotoxic functionality of the NK cell product. To this end, NK cells were isolated by an immunomagnetic negative selection (as described in Section “Materials and Methods”) and expanded *ex vivo* under feeder-cell free cultivation conditions addressing the impact of different cytokines. NK cells were cultured with IL-2^100^ (100 U/mL), IL-2^1000^ (1000 U/mL), IL-15 (10 ng/mL), IL-21 (25 ng/mL), combinations of these cytokines or IL-15 and a 3-day IL-21 boost (IL-15 + 21*_boost_*). Purity of the enriched NK cells was determined and cell numbers as well as viability were monitored over a period of 4–6 weeks (Figures 1A–D).

The average content of starting material after NK cell purification consisted of 89% NK cells. Purity of the CD3^neg^CD56^pos^ NK cells decreased slightly on day 3, but increased afterward during the culturing process. With protocols using IL-15, IL-15 + 21, and IL-15 + 21*_boost_*, the purity reached over 95% from day 10 on. In cultures containing exclusively IL-2 also CD3^pos^, cells expanded and diminished NK cell purity especially at late culture time points after 2–3 weeks (data not shown).

Remarkably, all protocols involving the addition of IL-15, such as IL-15 alone, IL-2^100^ + 15, and IL-15 + 21*_boost_*, led to a high increase in the number of NK cells over the first 10 days reaching a plateau until day 21. Permanent stimulation with IL-21 (IL-2^100^ + 15 + 21 and IL-15 + 21) also provoked rapid but less distinct expansion. In general, all expansion rates decreased slowly after 3 weeks of *ex vivo* cultivation (Figure 1A).

High levels of IL-2 (IL-2^1000^) evoked proliferation of NK cells during the first 6 days, but average expansion rates declined subsequently. Cultivation in the presence of low IL-2 levels (IL-2^100^) or IL-21 alone did not induce proliferation, instead NK cells died (Figures 1A–D).

Irrespective of donor-dependent differences, stimulation with IL-15, IL-2^100^ + 15, and IL-15 + 21*_boost_* performed better than all other stimulation protocols. Of note, permanent exposure to IL-21 dampened IL-15-driven expansion, while a short boost with IL-21 did not disturb proliferation of NK cells, but, in some cases, even increased the expansion rate. With the IL-15 + 21*_boost_* protocol, expansion rates ranged between 2- and 10-fold depending on the donor, exhibiting an average 4.5-fold increase on day 10 of culture (Figure 1C).

Percentages of viable cells decreased during the first days, but then recovered upon further stimulation (Figure 1B). Comparisons on day 10 of cultivation indicated significantly higher frequencies of viable cells in the product of IL-15-stimulated cells than in products obtained from expansion protocols with permanent addition of IL-21. Nevertheless, a short boost with IL-21 did not significantly affect cell viability compared to IL-15 mono treatment (Figure 1D).

### IL-21 Triggers a Mature Phenotype of NK Cells

**Figure 2:**
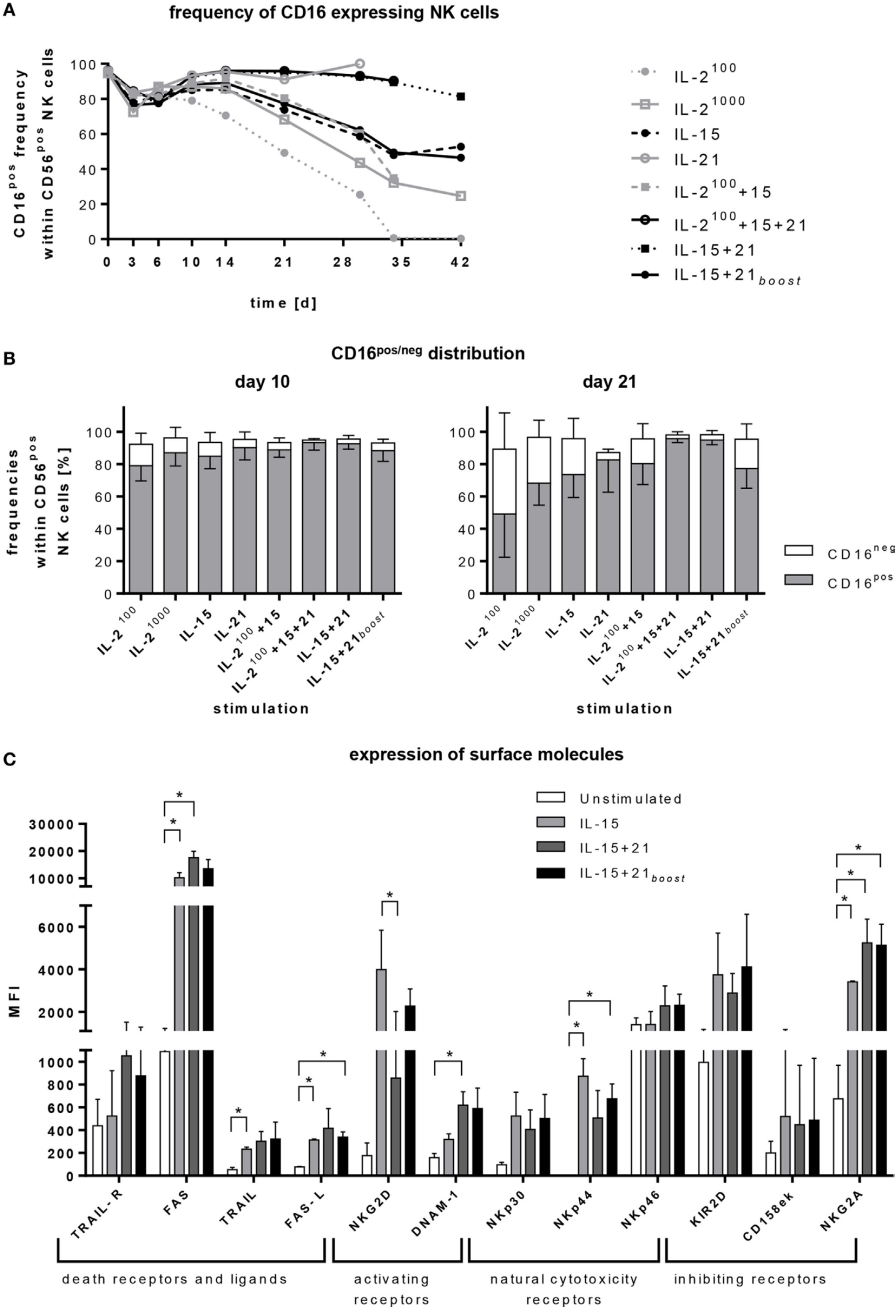
Regulation of surface markers. Purified CD56^pos^CD3^neg^ cells were cultivated for 4–6 weeks with different cytokines or cytokine combinations. **(A)** At indicated time points, frequencies of CD16^pos^ natural killer (NK) cells were assessed by flow cytometry. The graph represents means of 5–11 independent donors. **(B)** The graphs show the distribution of CD16^pos^ cells and their CD16^neg^ counterpart within CD56^pos^ NK cells on days 10 and 21. Bars represent mean values of 5–11 independent donors, lines indicate SDs. **(C)** Expression of various activating and inhibitory receptors after 6 days of cultivation is shown for selected protocols. Bars represent mean values of three independent donors, lines indicate SDs. Significant differences are indicated by asterisks (*p < 0.05, one-way ANOVA).

Next, we investigated changes in the phenotype and subset composition of NK cells triggered by cytokine-induced *ex vivo* expansion. To monitor their maturation state, NK cells were analyzed for the distribution of the CD56^high^CD16^neg^ and CD56^dim^CD16^pos^ subset over 4–6 weeks (Figures 2A,B). As all stimulation protocols induced an upregulation of the NK cell marker CD56, only a discrimination of CD16^pos^ and CD16^neg^ NK cells was implemented for further analysis of the maturation state.

Depending on the expansion protocol, the frequency of CD16^pos^ NK cells was reduced. Only protocols with permanent IL-21 exposure (IL-21, IL-15 + 21, and IL-2^100^ + 15 + 21) maintained a mature phenotype (Figure 2A). This effect became more pronounced the longer the *ex vivo* expansion endured (Figures 2A,B). In parallel with the decrease in mature phenotype, the proportion of less mature CD16^neg^ NK cells was increased (Figure 2B).

Furthermore, cytokine stimulation induced upregulation of activating and inhibitory receptors on the NK cell surface (Figure 2C). Interestingly, different cytokine combinations led to a significant increase of apoptosis inducing TRAIL or FAS ligand (FAS-L) in line with enhanced expression of TRAIL-R and FAS. We further observed that the presence of IL-21 had an additive effect with IL-15 regarding an enhanced surface expression of TRAIL, TRAIL-R, DNAM-1, FAS, FAS-L, NKp46, but also inhibitory NKG2A. For NKG2D, NKp30, NKp44 and inhibitory KIR2D and CD158 e/k (KIR3DL1/DL2), IL-21 counteracted the up-regulating effect of IL-15.

TRAIL, DNAM-1, NKp46, and NKG2A were rapidly upregulated upon short exposure to IL-21 if cells were pre-stimulated with IL-15 (IL-15 + 21*_boost_*) and reached expression levels similar to those of NK cells stimulated permanently with IL-15 and IL-21 (IL-15 + 21). By contrast, changes in the levels of other surface markers were less rapid, showing significant differences for cells stimulated following the different protocols.

Almost all CD16^neg^ NK cells expressed two of the three activating receptors, NKG2D, DNAM-1, and NKp44, only a small fraction of these cells expressed only one (NKG2D) and an even smaller fraction none. After 6 days of NK cell expansion, almost all CD16^neg^ NK cells co-expressed all three receptors (Figure S1 in Supplementary Material, left panels). Among the CD16^pos^ fraction, half of it expressed only NKG2D and half co-expressed NKG2D and DNAM-1. After expansion with IL-15 alone or an additional short-term IL-21 boost, on day 6 of culturing, half of the CD16^pos^ fraction expressed all three receptors, and half co-expressed two. Permanent exposure to IL-21 further increased the fraction of CD16^pos^ NK cells that co-expressed all three receptors. Altogether, no prominent difference in the co-expression of NKG2D, NKp44, and DNAM-1 was observed upon expansion with the three different protocols (Figure S1 in Supplementary Material, right panel).

To analyze the kinetics of the receptor expression induced by the different expansion protocols based on the use of IL-15 and/or additional IL-21, flow cytometry data were acquired at three time points, before stimulation, early at day 6, and late at day 18 (Figure S2 in Supplementary Material). Permanent presence of IL-21 diminished the expression of NKG2D, NKp30, and NKp44 on the NK cell surface. In contrast, it had hardly any influence, when applied after an IL-15-induced expansion phase. Expression of NKp46 was only slightly reduced by long-term IL-21 treatment. The higher expression level of DNAM-1, aroused by permanent presence of IL-21, stayed stable over time (Figure S2A in Supplementary Material).

Inhibitory receptors of the KIR family (KIR2D and CD158e/k/KIR3D) did not show any relevant changes during NK cell expansion, while NKG2A was strongly upregulated during NK cell expansion, independent from the cytokines used (Figure S2B in Supplementary Material).

Besides DNAM-1, expression of the adhesion molecules LFA-1 (CD11a) and 4-1BB (CD137) was addressed. Expression of LFA-1 was early increased under all tested cytokine conditions. In contrast, 4-1BB (CD137) was upregulated only after a longer culture period (Figure S2C in Supplementary Material). Accordingly, no prominent differences in conjugate formation were observed comparing the NK cells of all three tested cytokine expansion protocols (Figure S2D in Supplementary Material).

### Short Stimulation with IL-21 Increases NK Cell Cytotoxicity

Besides an increase in the yield of of NK cells, another aim of the *ex vivo* expansion is to obtain a therapeutic cell product that mediates optimal anti-tumor activity. In case of NK cell immunotherapeutics, cytotoxicity is a valuable indicator of functionality. Accordingly, cytotoxicity of cytokine-expanded NK cells was tested against targets such as the erythroleukemia cell line K562 and the RMS cell lines RD and RH30. The RD cell line represents the embryonal subtype of RMS while RH30 represents the alveolar subtype, which is more difficult to treat.

**Figure 3:**
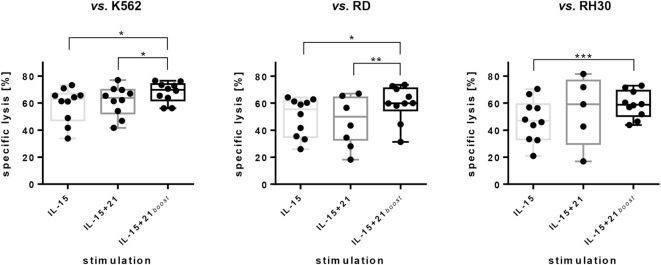
Cytotoxic capacity of *ex vivo* expanded natural killer (NK) cells against erythroleukemia cell line K562 and RMS cell lines RD and RH30. NK cells were expanded for 6 days utilizing the stimulation protocols indicated. Data are given as mean values and SDs obtained with E:T ratios of 10:1 after 5 h of co-incubation. Specific lysis was calculated by substracting spontaneous lysis values from frequencies of dead target cells. Spontaneous lysis was determined from target cells cultured without NK cells. Here, combined results from independent donors are shown (*n* = 10; *n* = 5 for NK cells expanded with IL-15 + 21 vs. RH30). Assays were performed in tripliates for each donor. Asterisks indicate significant differences (*p < 0.05, **p < 0.01, ***p < 0.005; vs. K562 and vs. RD: one-way ANOVA; vs. RH30: Student’s *t*-test).

A short boost with IL-21 increased the cytotoxic effect of NK cells toward all three cell lines compared to stimulation with IL-15 alone or permanent exposure to IL-21 (Figure 3). An increase in specific lysis of approximately 10% was achieved by an IL-21 boost for all three cell lines as compared to sole IL-15 stimulation.

Cytotoxicity of expanded NK cells decreased with longer culture periods and reduced from 60% on day 6 to 40% on day 10 and 14 (E:T = 10:1). Still cytotoxicity of continuously expanded cells was superior to cytotoxicity of NK cells that were cryoconserved after 6 days of cytokine expansion (Figure S3A in Supplementary Material).

Despite the decrease in CD16^pos^ NK cell numbers, no decrease of ADCC was observed with NK cells expanded with the IL-15 or IL-15 + 21*_boost_* protocols compared to NK cells expanded with the IL-15 + 21 protocol in an assay against ErbB2^pos^ RD cells supplemented with anti-ErbB2 antibody Trastuzumab. All expansion protocols resulted in an additional lysis of about 5 to 10% by ADCC at an E:T ratio of 10:1 (Figure S3B in Supplementary Material).

### The IL-21-Dependent Increase in NK Cell Cytotoxicity Is Based on Elevated Degranulation

To execute cytotoxicity upon activation by target cell recognition, one basic mechanism of NK cells is to degranulate and release cytolytic enzymes. In order to assess the impact of IL-21 to the cytolytic capacity and the underpinning mechanism, we stained for CD107a (lysosomal-associated membrane protein 1), which is located in the membranes of lytic granules and is expressed on the NK cell surface upon degranulation when lytic granules fuse with the outer membrane (47, 48).

**Figure 4:**
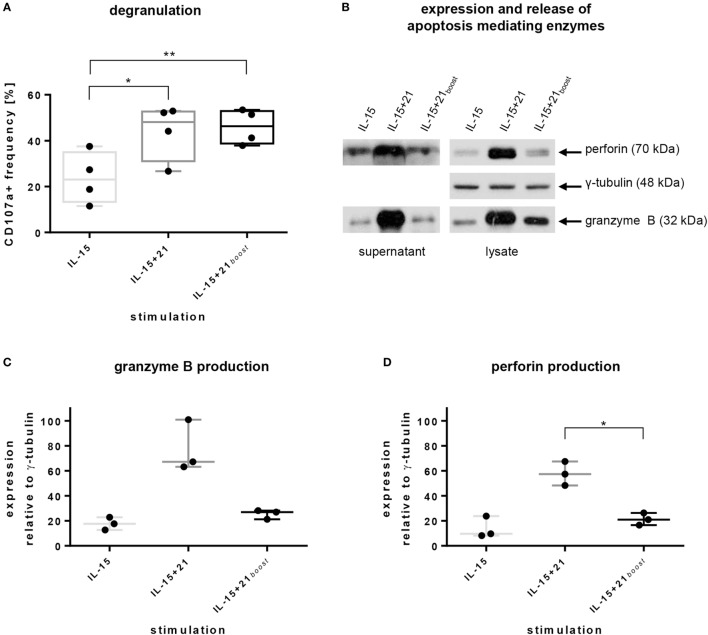
Natural killer (NK) cell degranulation capacity, intracellular production, and release of apoptosis-mediating enzymes induced by selected *ex vivo* expansion protocols. Purified NK cells were kept in culture for 6 days using indicated protocols. **(A)** CD107a levels on the surface of cells were acquired by flow cytometry. Bars express mean values from four independent donors, lines indicate SDs. **(B)** Exemplary immunoblot analysis of supernatants and lysates of NK cells from one representative donor after stimulation with selected *ex vivo* expansion protocols. Proteins were detected using monoclonal antibodies against human perforin, γ-tubulin, and granzyme B. **(C,D)** Mean values and SDs of intracellular production of granzyme B and perforin obtained from three independent donors. Values are calculated in relation to γ-tubulin expression. Asterisks indicate significant differences (*p ≤ 0.05, **p ≤ 0.01, one-way ANOVA).

Exposure to IL-21 during cultivation significantly increased the number of degranulating cells compared to IL-15 mono-treatment. This effect was rapidly induced after a short boost with IL-21. Permanent stimulation with IL-21 evoked similar results, suggesting that degranulation activity is maintained in the continuous presence of IL-21 (Figure 4A).

To further investigate if granule exocytosis is associated with cytolytic functionality, the expression of the pore-forming protein perforin and the serine protease granzyme B was addressed by immunoblotting. Indeed, expression and release of both proteins were induced strongly upon continuous stimulation with IL-21, but not subsequent to a short boost with IL-21 (Figures 4B–D). In particular, permanent presence of IL-21 in the culture provoked roughly a doubling of the relative expression of granzyme B and perforin in comparison to short-term or no IL-21 (Figures 4C,D).

In accordance with the slower induction of *de novo* production of granzyme B and perforin after short-term compared with continuous exposure to IL-21, apoptosis-inducing enzymes were released to a lesser extent despite similar degranulation levels were observed under both conditions (Figures 4A,B).

To further evaluate if this increase of degranulation was correlated with an increase of PI3K pathway signaling, we measured the phosphorylation of AKT and ERK1/2. Both were upregulated using IL-15 for expansion and even more with additional IL-21 (Figure S4 in Supplementary Material).

### IL-21 Exposure Increases Cytokine Release by NK Cells

**Figure 5:**
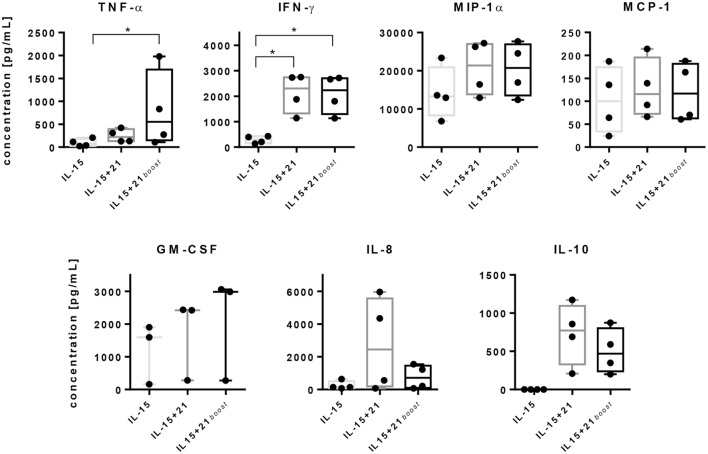
Release of cytokines by purified natural killer (NK) cells upon stimulation with selected expansion protocols. Purified NK cells were cultured using different stimulation protocols. On day 6, release of tumor necrosis factor (TNF)-α, IFN-γ, macrophage inflammatory protein (MIP)-1α, monocyte chemoattractant protein (MCP)-1, granulocyte-macrophage colony-stimulating factor (GM-CSF), IL-8, and IL-10 were assayed by cytometric bead array analyses. Graphs show mean values and SDs obtained from four independent donors, except GM-CSF, which was measured for three independent donors. Asterisks indicate significant differences (*p < 0.05, one-way ANOVA).

Natural killer cells can mediate direct cytotoxicity but also have an immunoregulatory function. The latter is achieved by secretion of cytokines. Hence, we analyzed the release of different cytokines after *ex vivo* expansion of NK cells using cytometric bead arrays (Figure 5).

Secretion of TNF-α was induced slightly by IL-15 and increased significantly upon stimulation with IL-21. Remarkably, after short-term exposure to IL-21, the TNF-α level was even higher in comparison with a continuous exposure to IL-21. A similar effect was observed for GM-CSF, although to a lesser extent. IFN-γ concentrations were significantly elevated with both protocols that utilized IL-21 causing a 10-fold increase in IFN-γ levels compared to IL-15 alone. Similarly, MIP-1α was secreted to a higher extent in the presence of IL-21 compared with IL-15 alone, independent of the duration of administration (Figure 5).

Levels of MCP-1 were similar for all three stimulation protocols, while IL-8 release was gradually more induced by IL-21 as compared to IL-15 alone and was further increased depending on the duration of IL-21 exposure. Also the levels of anti-inflammatory IL-10 were increased depending on the duration of IL-21 presence. In contrast, IL-15 application alone almost prevented secretion of IL-10 by NK cells (Figure 5).

### IL-21-Boosted Stimulation Enables *In Vivo* Cytotoxicity of NK Cells following Adoptive Transfer in a Xenograft Model

**Figure 6:**
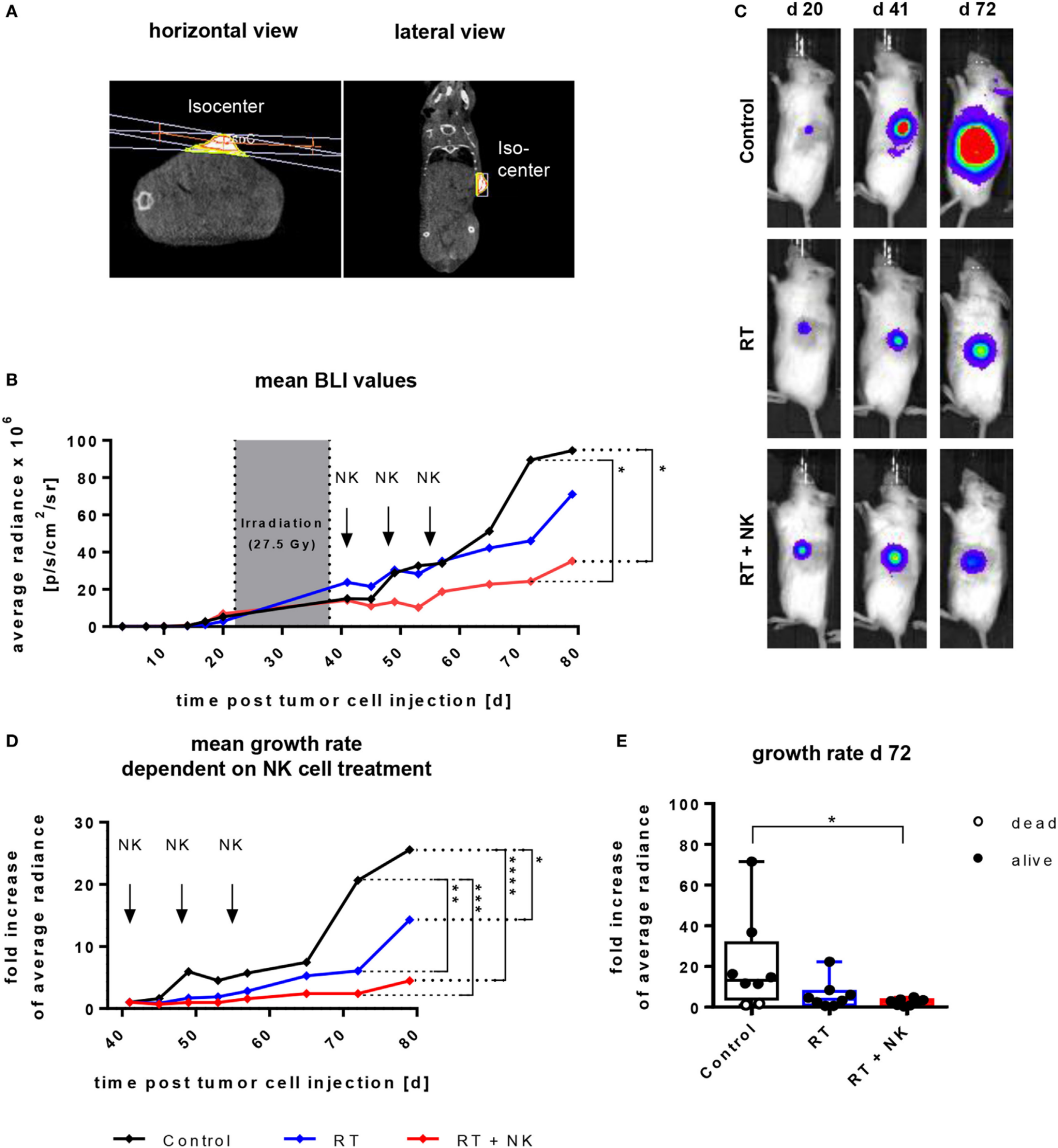
Adoptive transfer of *ex vivo* IL-15-expanded and IL-21 boosted natural killer (NK) cells into RMS-bearing NSG mice subsequent to radiotherapy (RT). Mice were subcutaneously injected with 10^5^ RD cells and 3 weeks later, tumor-bearing mice underwent local RT, followed by adoptive transfer of 10^7^ NK cells by three weekly injections. NK cells were generated using the IL-15 + 21*_boost_* protocol. **(A)** Exemplary CT-image guided planning of high precision tumor irradiation with a two-field geometry and RT isocenter presented in a horizontal and lateral view. **(B)** Time course of tumor growth, showing mean average radiance values from eight mice per group. The gray area indicates the period of RT, arrows indicate time points of NK cell administration. Significant differences are given as asteriks (*p ≤ 0.05). **(C)** Pictures from bioluminescence imaging (BLI) of exemplary mice from each treatment group obtained before starting treatment (day 20), after termination of RT (day 41), and at the end of the experiment (day 72). **(D)** Kinetics of relative tumor growth rates normalized to tumor size at onset of NK cell treatment and **(E)** at day 72 after tumor inoculation. Displayed are mean values from eight mice per group and single values for each mouse **(E)**. Dead mice are shown as empty circles, asterisks indicate significant differences (*p ≤ 0.05, **p ≤ 0.01, ****p ≤ 0.001, two-way ANOVA).

Given that NK cells cultured with the IL-15 + 21*_boost_* protocol exhibited the highest expansion rate and cytotoxic activity toward different target cells, we finally aimed to validate the *in vivo* effectiveness of NK cells expanded with the two-phase protocol in a RMS xenograft model. To this end, NOD/SCID/IL-2Rγc^−^ (NSG) mice were subcutaneously injected with the RD^GFP/Luc^ cell line and after establishing tumors, mice were locally irradiated by an image guided high precision local RT (Figure 6A). Subsequently, adoptive transfer of IL-15 + 21*_boost_* continuously expanded NK cells was performed by three consecutive injections of 10^7^ NK cells. Tumor burden was monitored by caliper measurement (not shown) or BLI analysis of eight mice per group over 79 days (Figure 6B). Animals treated with RT monotherapy displayed a significant decrease in luciferase intensity indicating a growth retardation (Figures 6C,D). Following combined RT and NK cell therapy, the cytostatic effect was even more pronounced as compared to untreated or irradiated tumor-bearing controls (Figures 6B–E).

## Discussion

Immunotherapy represents a promising approach against malignant diseases. In this context, NK cells have been explored for several years because they exhibit prevailing anti-tumor activity (9, 49–54). A major advantage of NK cell-based immunotherapy is the possibility to employ these cells in an allogeneic or haploidentical setting (6, 55, 56) without causing, or even preventing, GvHD (37–41). Numerous attempts have been made to expand NK cells efficiently for adoptive cell transfer focusing on different aspects such as high yields, efficient activation, cytotoxic potential, and/or good manufacturing practice (GMP) adequacy (27). Unfortunately, there are only limited publications available that state expansion rates after stimulating NK cells with cytokines only. Especially protocols using IL-21 on purified NK cells are quite rare. Wendt et al. did not mention absolute NK cell numbers but found an increased proliferation upon an IL-2 + 21 expansion over 72 h as shown by [^3^H] thymidin incorporation. Researchers around Koehl et al. repeatedly reported expansion rates between four and five times after 12–14 days of culturing with high dose IL-2^1000^. These results are similar to ours obtained with IL-15 or IL-15 + 21*_boost_*, although our IL-2^1000^ expansion did not exceed a three times increase in our tests. Compared to that, protocols allowing additional accessory PBMCs resulted in an expansion rate of 23-fold by IL-15 (57), while under addition of IL-21 only a 3.7-fold expansion rate was reached (23). Addition of gene modified feeder cells resulted in several hundreds of multiplications. When IL-21 was added to such culturing conditions, an expansion of up to 2.7 × 10^11^ was reached after 46 days (24). The use of membrane bound IL-21 and 4-1BB ligand still led to an expansion of more than 10^5^-fold (26).

In order to avoid contamination by possible GvHD causing cell subsets or genetically modified, tumor-derived feeder cells, we used purified NK cells for defining efficient expansion protocols. The major goal of the present study was to establish a feeder-cell free protocol for efficient expansion of primary NK cells and the improvement of their cytolytic capability to target highly aggressive and radiation-resistant RMS.

Natural killer cells were purified from buffy coats and stimulated with selected common γ-chain cytokines in different combinations. In the case of IL-2 stimulation, this study confirmed that the number of NK cells decreased under a low level of IL-2 (100 U/mL) and increased only slightly upon administration of a high concentration of IL-2 (1000 U/mL) (Figure 1A) (14). However, as low IL-2 levels were shown to reduce CD3^pos^ T cell outgrowth from contaminating cells remaining after NK cell purification (58), combinations of a low IL-2-dosis with other cytokine stimulants more specifically improve NK cell expansion (59, 60). Of note, all IL-15-based expansion protocols, which were tested in this study, induced an increase in the amount of NK cells in the early expansion phase. In contrast, long-term presence of IL-21 negatively influenced NK cell proliferation capacity, while a short-time IL-21 boost up to 3 days enhanced proliferation and NK cell cytotoxicity (Figures 1A,C and 3). In fact, the effects of IL-21 in NK cell proliferation are controversial. Immobilized or membrane-bound IL-21 has a positive effect on NK cell expansion (25, 61). Also, the longer survival of human NK cells following combined IL-21 and IL-2 stimulation has been reported (18). Conversely, an IL-21-induced apoptosis resulting in limited life span of NK cells has been described (20, 62). Overall, IL-21 appears to play an essential role in NK cell activation and cytotoxicity (21, 62).

Here, we observed a decline in the viability of NK cells after long-term exposure to IL-21 when added to other cytokines. Based on this observation, we developed a two-phase protocol first using IL-15 for the optimal expansion of NK cells, complemented by short-term addition of IL-21 to support proliferation and enhance the cytotoxic potential (IL-15 + 21*_boost_*; Figures 1A,B and 3). Regarding the cytokine-induced expansion of NK cells, it is important to note that most published and also our own results demonstrate a strong donor-dependent variability. It will be of high interest and potential clinical relevance to address and better understand underlying mechanisms of a donor-dependent variation in cytokine-induced NK cell response.

Cytokine stimulation and *ex vivo* expansion modulate the phenotype and function of NK cells (52). CD56 surface expression has been reported to be reduced during the purification process, down to complete absence of CD56 from the cell surface, but to be upregulated upon *in vitro* stimulation (63). Accordingly, we also found an increased expression of NK cell activating receptors upon cytokine stimulation (Figure 2). The CD16-expressing NK cell population was diminished during stimulation with IL-2 or IL-15 (Figure 2A), as previously reported to be mediated by metalloproteinase ADAM17 (64–66). In contrast, IL-21 maintained surface expression of CD16, hence, increasing the frequency of the more mature CD16^pos^ NK cell population (Figure 2B).

In parallel with the changes in CD16 expression, apoptosis-mediating receptors and ligands such as TRAIL, FAS, and FAS-L were upregulated upon cytokine-induced expansion. In this respect, we observed a marked increase in FAS expression triggered by all protocols (Figure 2C), confirming recent reports (67, 68). Usually, this mechanism is employed for the killing of tumor cells but also plays a part in the achievement of lymphocyte homeostasis following immune responses against infections (69). Herein, we observed TRAIL, FAS-L, DNAM-1, and NKp46 to be expressed strongly on the NK cell surface under IL-21 stimulation when compared with IL-15 stimulation alone (Figure 2C). In contrast, the expression of NKG2D, NKp30, and NKp44 was reduced under the influence of IL-21. It has been reported that NKp44 is downregulated by IL-21 treatment post-IL-15 stimulation (70, 71). Also, a decreased expression of NKp44 and NKG2D was attributed to IL-21-mediated inhibition of DAP10 and DAP12 (71, 72). In contrast, our results showed that the downstream signaling of DAP10 *via* the PI3K pathway was activated by IL-15, and even increased upon usage of IL-21 for NK cell expansion (Figure S4 in Supplementary Material). Upregulation of AKT phosphorylation is known to correlate with proliferation and cell survival. The even more activated ERK1/2 is associated with NK cell cytotoxicity, mediated by perforin and granzyme B mobilization (73, 74).

The maturation and activation state of NK cells in the final product is important, but the ability to carry out cytolytic functions is crucial. In line with that, we analyzed specific killing of the K562 erythroleukemia cell line as well as rhabdomyosarcoma cell lines RD and RH30. We observed an increase of cytotoxicity upon using the IL-15 + 21*_boost_* protocol compared to IL-15 mono-treatment (Figure 3). For repeated NK cell applications, we tested continuously expanded cells and compared their cytotoxicity to that of cryopreserved cells. In line with reports on IL-15-stimulated NK cells (75), in our study, we observed a reduced cytotoxic capacity of cryopreserved cytokine-stimulated compared to continuously expanded NK cells (Figure S3A in Supplementary Material) and therefore performed analysis with the latter if several rounds of NK cell application were necessary. Another advantage of NK cell expansion using a feeder cell free IL-15 + 21*_boost_* protocol will be that it easily fulfils the GMP criteria required for clinical application. Previous reports showed that IL-15-stimulated NK cells may attack various RMS cell lines more efficiently than unstimulated NK cells. Moreover, these investigations indicated that DNAM-1 and NKG2D may comprise initiators of cytotoxicity for resting NK cells, while killing by IL-15-stimulated NK cells involves additional factors including NKp30 and NKp46 (76). In our study, IL-21 enhanced NK cell cytotoxicity compared to IL-15 mono-treatment, increased DNAM-1 but reduced NKG2D expression. Although, LFA-1, among others, has been shown to be involved in the degranulation process (77) and we observed an increase in degranulating cells after prolonged IL-21 exposure or an IL-21 boost, we did not notice any difference in the expression levels of CD11a between the different stimulation protocols and also no prominent differences in the ability to form target cell conjugates (Figures S2C,D in Supplementary Material).

As previous reports showed that IL-21 induced killing can be independent from death receptor expression (62), but mediated by perforin (78), we investigated the release of lytic granules as one main mechanism mediating NK cell cytotoxicity. Indeed, IL-15 + 21*_boost_*-stimulated NK cells showed an increased exposure of CD107a (Figure 4A), which is known to be an indicator of NK cell activity and degranulation (48). We further found that IL-21 strongly induced synthesis and release of granzyme B and perforin, but only after prolonged exposure. This is in accordance with reports on an increased production of perforin and IFN-γ in cytotoxic CD8^pos^ T cells from HIV patients upon IL-21 stimulation (79). Moreover, upregulation of intracellular perforin was reported for CD56^pos^ cells from HIV-infected individuals (80), from stimulated PBMCs (18) and from patients with malignant melanoma upon IL-21 stimulation (81). Moreover, the increased activation of AKT and ERK signaling that we observed with IL-21 has been reported to be accompanied by intracellular perforin and granzyme B redirection (73).

Although cytokine release follows pathways distinct from lytic granule exocytosis, both mechanisms often are regulated similarly (82). In parallel to the release of lytic granules, secretion of TNF-α and IFN-γ was elevated after IL-21 stimulation (Figure 5). In the context of an immunocompetent host, NK cells not only contribute to the immune response by direct cytotoxicity but also as mediators at an intersection between innate and adaptive immunity (83, 84). Surprisingly, secretion of IL-10 was strongly induced by IL-21, but not by IL-15. As IL-10 is reported to play a crucial role in immune suppression, its function following IL-21 stimulation remains to be evaluated. Nevertheless, opposing effects were reported for IL-12, which was shown to activate NK cells and trigger pro-inflammatory T cell responses (85, 86), but also to induce IL-10 secretion (87).

Finally, we addressed the *in vivo* anti-tumor efficacy of IL-15 + 21_boost_ expanded NK cells if combined with ionizing radiation in a RMS xenograft model. Adoptive NK cell immunotherapy in combination with RT has been addressed only in a few studies so far. Ames et al. recently reported that NK cell inoculation was effective at targeting cancer cells with a stem cell phenotype from a variety of solid malignancies, which was most effective when combined with RT before application (88). It has further been shown that a combined transfer of IL-12 + 15 + 18 activated NK cells and high dose (5 Gy) synchronous irradiation resulted in a growth retardation of RMA-S lymphoma and B16-RAE-1ε tumor cells (84). Consequently, in our proof of principle approach, we performed a multimodal treatment with a preceding local RT followed by adoptive transfer of IL-15 + 21_boost_ expanded NK cells. Here, we confirmed that combined NK cell immunotherapy and RT was superior to RT monotherapy in terms of growth retardation, which reaches a level of significances at 7.5 weeks. Moreover, for that purpose, a mouse CT-image guided and individually planed local irradiation procedure was applied using a SARRP with a fractionated daily 2.5 Gy RT protocol that to higher extent reflects the clinical situation where patients were treated with single 1.8 to 2 Gy fractions. Notably, combined modality treatment failed to cure the tumor burden (Figures 6B,D) indicating the necessity for advanced protocols with several repeated NK transfers or additional *in vivo* cytokine injections to further increase therapeutic efficacy. This will be addressed in future investigations.

In conclusion, we presented an optimized protocol for *ex vivo* NK cell expansion that combines the positive effects of both IL-15 and IL-21 on proliferation and activity of NK cells and that offers an ideal expansion protocol also under GMP conditions due to the absence of feeder cells. Additionally, our findings demonstrate the high *in vitro* and *in vivo* antitumor efficacy of IL-15 + 21*_boost_* expanded NK cells, which may become useful for the development of innovative combined modality treatment strategies for radiation-resistant RMS.

## Ethics Statement

This study was carried out in accordance with the recommendations of the Ethics Committee of the Goethe University Frankfurt (Frankfurt, Germany). All subjects gave written informed consent in accordance with the Declaration of Helsinki. This study was carried out in accordance with the recommendations of German Animal Welfare Act. The protocol was approved by the government committee (Regierungspräsidium Darmstadt, Darmstadt, Germany).

## Author Contributions

JW, EU, FR, and PB designed the project. JW, VP, JB, AW, SH, and BB performed experiments and analyzed data. JW, EU, WW, FR, PB, CB, TK, and SH discussed data. JW and EU wrote the manuscript with support from all other co-authors. All authors agreed to be accountable for the content of the work.

## Conflict of Interest Statement

The authors declare that the research was conducted in the absence of any commercial or financial relationships that could be construed as a potential conflict of interest.
