# Supplementary material for: A Two-Phase Expansion Protocol Combining Interleukin (IL)-15 and IL-21 Improves Natural Killer Cell Proliferation and Cytotoxicity against Rhabdomyosarcoma
Source: Front Immunol. 2017 Jun 12;8:676. doi: 10.3389/fimmu.2017.00676 (PMC5466991; doi:10.3389/fimmu.2017.00676)
Supplement: Supplementary file 1 [file data_sheet_1.docx]

**Supplemental Data**

# A Two-Phase Expansion Protocol Combining IL-15 and IL-21 Improves NK Cell Proliferation and Cytotoxicity Against Rhabdomyosarcoma

Juliane Wagner, Viktoria Pfannenstiel, Anja Waldmann, Judith W. J. Bergs, Boris Brill, Sabine Hünecke, Thomas Klingebiel, Franz Rödel, Christian J. Buchholz, Winfried S. Wels, Peter Bader, Evelyn Ullrich


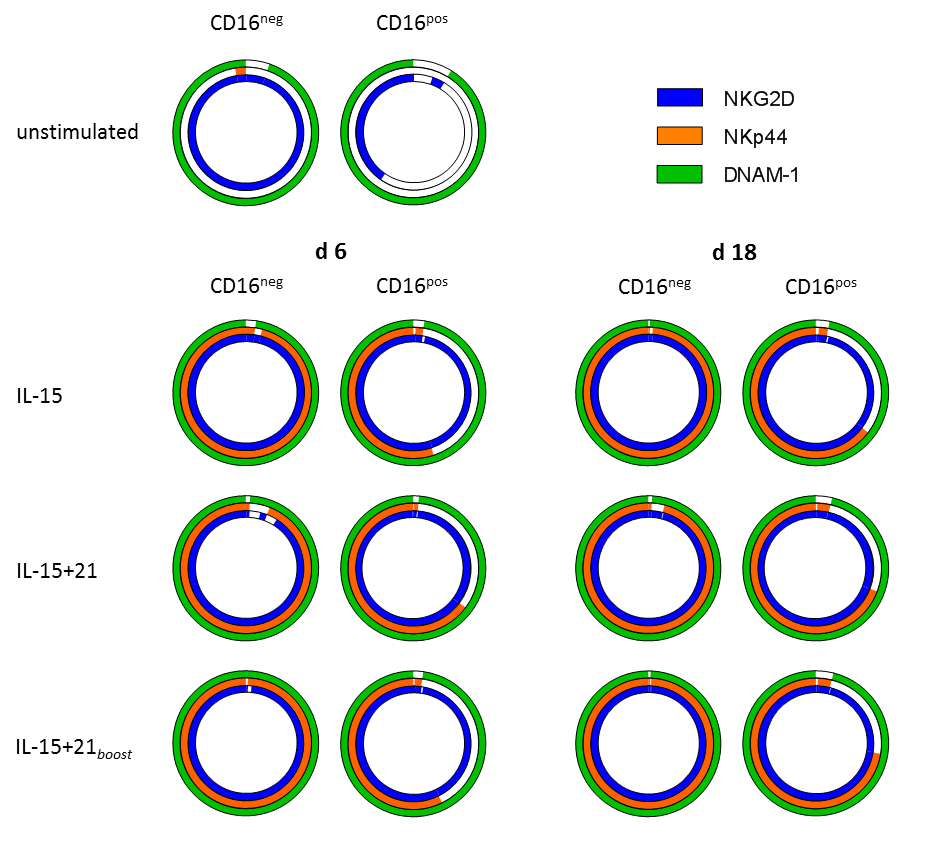


**Suppl. Figure 1 IL-21 slightly enhanced the co-expression of NKp44 with NKG2D and DNAM-1**  Unstimulated NK cells and NK cells expanded using the protocols IL-15, IL-15+21 or IL-15+21*_boost_* were stained for the activating receptors NKG2D, NKp44 and DNAM-1 in one common panel to determine co-expression of these receptors on CD16^neg^  and CD16^pos^ NK cells. Data were obtained from one donor.


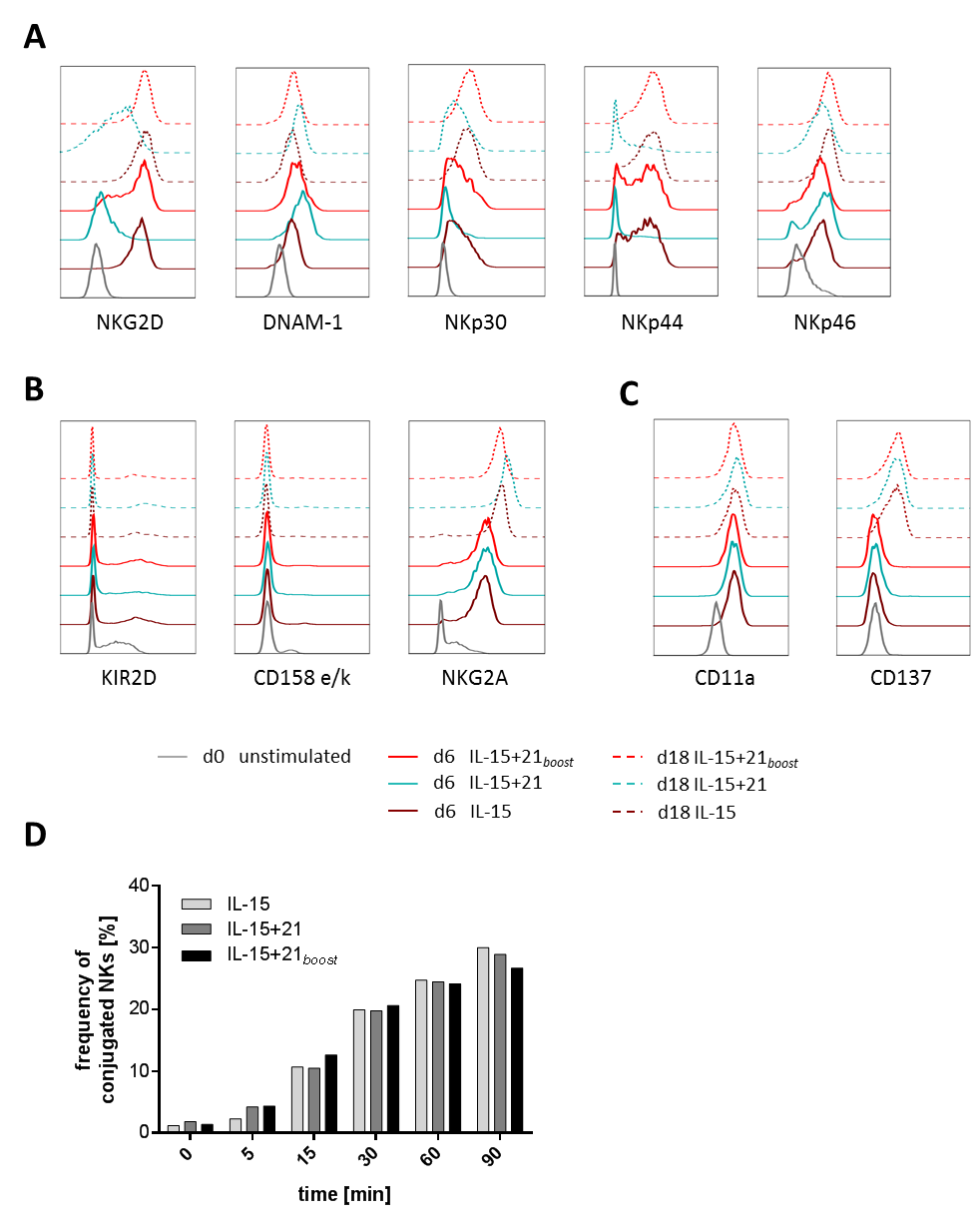


**Suppl. Figure 2 Time dependent changes in the NK cell phenotype and NK cell ability for conjugate formation after expansion** NK cells were expanded for up to 18 days using the three different protocols, IL-15, IL-15+21, IL-15+21*_boost_* and stained for activating receptors (**A**), inhibitory receptors (**B**) and adhesion molecules (**C**) on day 0, before expansion and during expansion at an early and a late time point, on day 6 and day 18. For most activating receptors slightly elevated expression levels at day 18 were shown compared to day 6. Also inhibitory NKG2A was increased, while receptors of the KIR2D and KIR3D family stayed stable. **D** Besides adhesion receptor expression, conjugate formation between NK cells and target cells was addressed in a time course assay ranging from 0 to 90 min co-incubation. At short co-incubation times conjugate formation is enhanced with IL-15+21*_boost_* expanded NK cells, while IL-15 expanded cells show higher frequencies of conjugated cells after longer co-incubation times. Graphs display results from single donors.


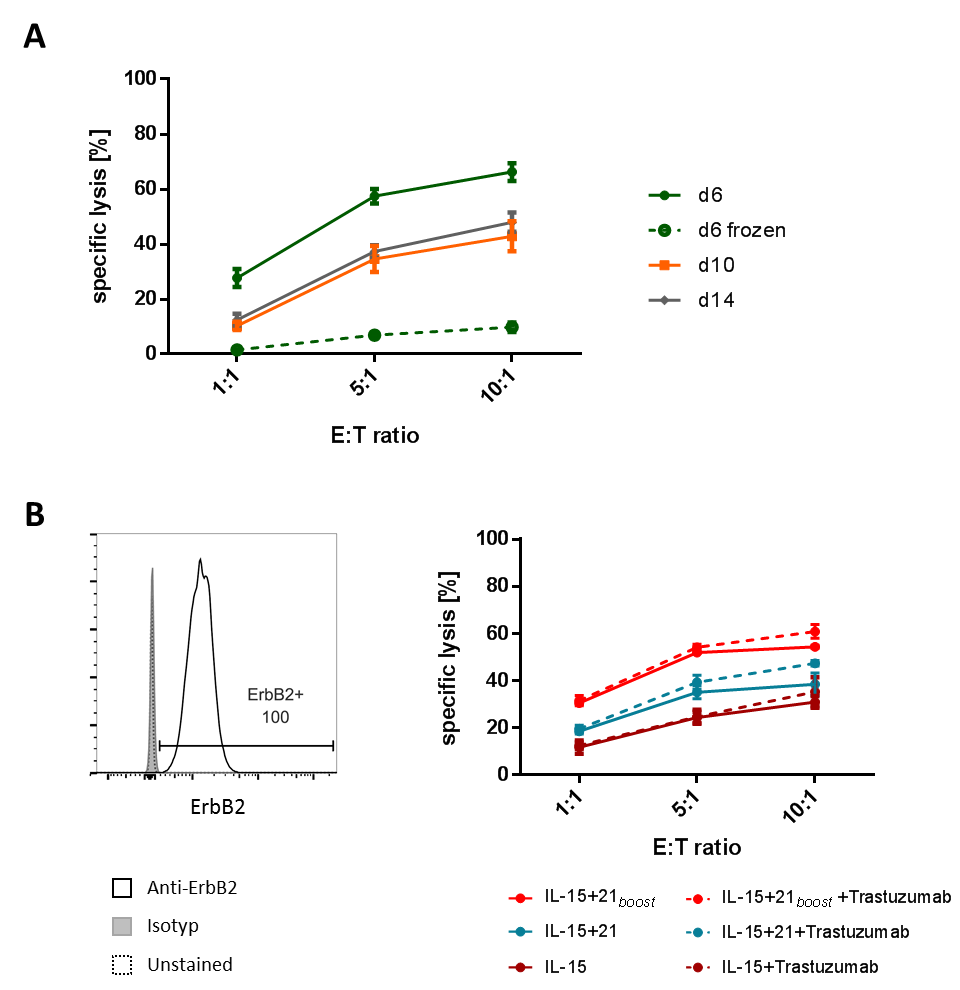


**Suppl. Figure 3 Influence of culture process on direct and antibody dependent NK cell cytotoxicity
A** NK cells were expanded using the IL-15+*21_boost_* protocol and cytotoxicity was acquired on day 6, 10 and 14. Additionally, NK cells expanded for 6 days were harvested, frozen and thawed after two weeks (d6 frozen). Cytotoxicity was diminished with longer culture periods, but still superior compared to frozen NK cells. The graph displays triplicates obtained from NK cells that were isolated from one donor. **B** Left graph shows the ErbB2 expression by RD cells that were used to analyze ADCC of NK cells. Addition of Trastuzumab to NK cells expanded for 10 days, slightly enhanced their cytotoxicity against ErbB2^pos^ RD cells. No prominent differences in the enhancement of cytotoxicity were observed between the three protocols. The graph represent triplicates obtained from one donor.

**
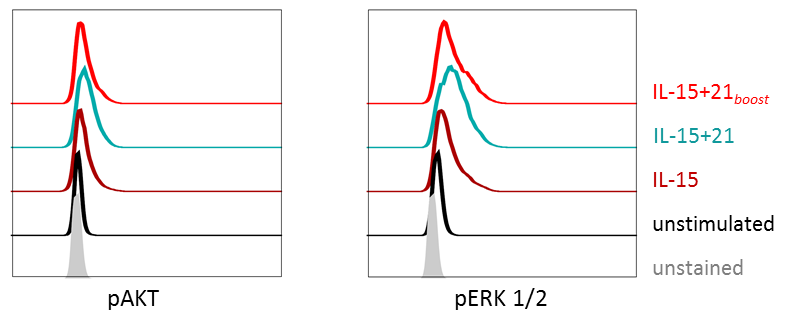
**

**Suppl. Figure 4 IL-21 enhanced activation of protein kinase AKT and extracellular regulatory kinase ERK1/2** NK cells were expanded using either of the three protocols, IL-15, IL-15+21 or IL-15+21*_boost_* and on day 6 stained intracellularly for the phosphorylated version of PI3K pathway downstream molecules AKT and ERK1/2. Both were elevated during expansion, but IL-21 enhanced activation of AKT and ERK1/2 slightly more compared to IL-15 alone.
